# Celecoxib Combined with Tocilizumab Has Anti-Inflammatory Effects and Promotes the Recovery of Damaged Cartilage via the Nrf2/HO-1 Pathway In Vitro

**DOI:** 10.3390/biom14121636

**Published:** 2024-12-20

**Authors:** Miyako Shimasaki, Shusuke Ueda, Masaru Sakurai, Norio Kawahara, Yoshimichi Ueda, Toru Ichiseki

**Affiliations:** 1 Department of Pathology 2, Kanazawa Medical University, Daigaku 1-1, Uchinada-machi, Kahoku-gun 920-0293, Japan; miya0807@kanazawa-med.ac.jp; 2 Department of Orthopaedic Surgery, Kanazawa Medical University, Daigaku 1-1, Uchinada-machi, Kahoku-gun 920-0293, Japan; adeu221@kanazawa-med.ac.jp (S.U.);; 3 Social and Environmental Medicine, Kanazawa Medical University, Daigaku 1-1, Uchinada-machi, Kahoku-gun 920-0293, Japan; 4 Department of Pathology, Keiju Medical Center, 94, Tomioka-machi, Nanao 926-0816, Japan; 5 Division of Translational Research, Department of Life Science, Medical Research Institute, Kanazawa Medical University, Daigaku 1-1, Uchinada-machi, Kahoku-gun 920-0293, Japan

**Keywords:** antioxidant, cartilage damage, celecoxib, chondrocytes, heme oxygenase-1, oxidative stress, tocilizumab, Nrf2

## Abstract

Inflammation and oxidative stress are crucial for osteoarthritis (OA) pathogenesis. Despite the potential of pharmacological pretreatment of chondrocytes in preventing OA, its efficacy in preventing the progression of cartilage damage and promoting its recovery has not been examined. In this study, an H_2_O_2_-induced human OA-like chondrocyte cell model was created using H1467 primary human chondrocytes to evaluate the efficacy of interleukin (IL)-6 and cyclooxygenase (COX)-2 inhibitors (tocilizumab and celecoxib, respectively) in the prevention and treatment of cartilage damage. H_2_O_2_ significantly elevated the IL-6, COX-2, and matrix metalloproteinase (MMP)-13 levels. Although monotherapy decreased the levels, nuclear shrinkage and altered cell morphology, similar to those in the H_2_O_2_ group, were observed. The expression of these factors was significantly lower in the combination therapy group, and the cell morphology was maintained. Moreover, the nuclear factor erythroid 2-related factor 2 (Nrf2) pathway was activated, and levels of the antioxidant protein heme oxygenase-1 (HO-1) were increased, especially in the combination group, indicating an anti-inflammatory effect. The treatment groups, particularly the combination group, demonstrated increased cell viability. Overall, the drug combination exhibited superior efficacy in preventing the progression of cartilage damage and promoted its recovery compared with the monotherapy. Given that the drugs herein are already in clinical use, they are suitable candidates for OA treatment.

## 1. Introduction

Osteoarthritis (OA) is a common chronic inflammatory joint disease that causes pain and disability not only in the elderly but in young people [1]. In the present aging society, the incidence of OA is increasing, and preventing disease progression and relieving pain assume greater importance. However, efforts to develop effective disease-modifying drugs for OA treatment have been unsuccessful despite years of research [2]. Advanced OA often requires surgical interventions, such as joint replacement [3]. Although prostheses have greatly improved outcomes, long-term outcomes and potential requirement for multiple surgeries, including revision surgeries in younger patients, remain significant causes for concern. Therefore, routine measures for preventing the progression of OA and reducing inflammation are needed for effective time-saving in OA treatment and prolonging the period from the onset of OA to surgery. In recent years, proinflammatory molecules, such as interleukin (IL)-6 and cyclooxygenases (COX)-2 [4,5,6], have been identified as important etiological factors for OA. The involvement of oxidative stress in joint destruction in OA has also been reported [7,8].

Inflammatory factors, such as inflammatory cytokines [IL-1β, IL-6, IL-15, IL-17, IL-18, and tumor necrosis factor (TNF)-α], prostaglandins, and reactive oxygen species (ROS) released by chondrocytes, synovial cells, and other cells, induce destruction of cartilage structure and function [9,10]. However, despite years of research, the exact etiology of OA remains unknown [11,12]. Generally, cartilage damage caused by inflammation and oxidative stress leads to further inflammation and deterioration. Inflammation also causes pain, and persistent pain induces central sensitization [13,14]. Thus, a vicious cycle of cartilage damage and pain is operative in OA. Therefore, suppressing the inflammatory cytokines involved in the development of OA and increasing the levels of antioxidant factors are important for preventing OA progression and controlling pain.

Recently, the use of mesenchymal stem cells and other cells for prevention of OA progression and pain control has been reported; however, the use of mesenchymal stem cells as a general treatment in clinical practice is still not practicable. Therefore, drugs possessing the ability to inhibit cartilage damage in clinical practice need to be discovered. In this context, the use of existing pharmaceuticals could be an effective method for preventing cartilage damage. In vitro investigation of the treatment of cartilage damage caused by IL-1β via pretreatment with COX-2 inhibitors and glucosamine has been reported [15]. Although this pretreatment study revealed the prophylactic efficacy of these drugs in OA cartilage injury models, their therapeutic effect on damaged cartilage cells remains uncertain.

In the present study, we investigated the therapeutic effects of clinically used IL-6 and COX-2 inhibitors (tocilizumab and celecoxib, respectively) on damaged chondrocytes, in addition to the effects of pretreatment with these inhibitors, as a monotherapy, or in combination.

## 2. Materials and Methods

### 2.1. Establishment of Preventive and Therapeutic Model Using a Human Osteoarthritis-like Chondrocyte Injury Model

**Figure 1:**
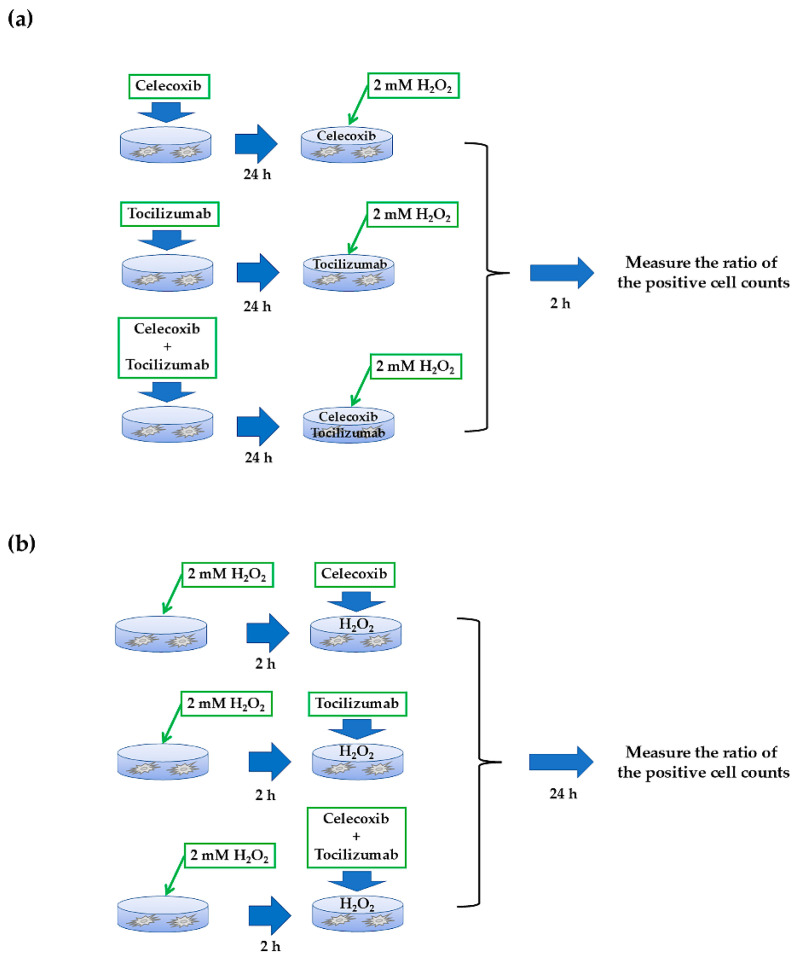
Schematic of the experimental platform. (**a**) Assessment of the preventive potential of mono- and combination therapy on H_2_O_2_-induced chondrocyte damage. (**b**) Assessment of therapeutic potential of mono- and combination therapy on H_2_O_2_-induced chondrocyte damage. (For details, see Section 2).

Human chondrocytes (donor: H-1467 male, age 56, normal) are primary human chondrocytes harvested from the knee joint and were purchased from Articular Engineering (Northbrook, IL, USA). Human chondrocytes were maintained as subconfluent monolayer cultures in a chondrocyte growth medium (Articular Engineering) supplemented with 10% fetal bovine serum. For analyses, chondrocytes were cultured to 70% confluence at 37 °C in an atmosphere of 20% O_2_ and 5% CO_2_. The cells were treated with 100 μM H_2_O_2_ (WAKO, Tokyo, Japan) for 30 min or 2 h to establish an in vitro OA chondrocyte model [16]. First, the cells were treated with the inflammation inhibitors, 10 μM celecoxib (Selleck Chemicals, Houston, TX, USA) [17] and 1.0 μg/mL tocilizumab (Selleck Chemicals) [18] for 24 h, followed by 100 μM H_2_O_2_ (prevention group) to evaluate the preventive effect of the inhibitor (Figure 1a). Next, damaged chondrocytes were stimulated with 100 μM H_2_O_2_ for 2 h and treated with celecoxib and tocilizumab alone or in combination in the presence of H_2_O_2_ for 24 h (treatment group) (Figure 1b). For control, cells were cultured in a medium without H_2_O_2_ and inhibitors under 20% normoxia (control group) or in a medium containing H_2_O_2_ (inflammation group).

### 2.2. Immunostaining for IL-6, COX-2, Matrix Metalloproteinase (MMP-13), Nuclear Factor-Erythroid 2-Related Factor 2 (Nrf2), and Heme Oxygenase (HO)-1

Cultured cells were fixed in 4% paraformaldehyde, washed with phosphate-buffered saline (PBS), and permeabilized with 0.3% Triton X-100 in PBS. Nonspecific binding was blocked by incubating the cells with 10% bovine serum albumin (DakoCytomation, Santa Clara, CA, USA) in PBS for 15 min. They were then incubated with anti-IL-6 (1:300; Proteintech, Rosemont, IL, USA), anti-COX-2 (1:500; Proteintech), anti-MMP-13 (1:200; Proteintech), anti-Nrf2 (1:200; Proteintech), or anti-HO-1 (1:200; Proteintech) antibodies for 2 h. The cells were then incubated with Alexa 488-labeled secondary antibodies (Thermo Fisher Scientific, Waltham, MA, USA) for anti-Nrf2, anti-HO-1, and anti-COX-2 or Alexa 594-labeled secondary antibodies for anti-IL-6. The cells were then incubated with 4′,6-diamidino-2-phenylindole (DAPI) for 30 min. After washing, a prolonged diamond antifade mountant (Thermo Fisher Scientific) was added and coverslips were mounted. Images were captured using Zeiss-LSM710 (Zeiss, Baden-Württemberg, Germany) and BZ-X700 (Keyence, Tokyo, Japan) microscopes. The experiment was repeated three times independently. The expression of each protein in chondrocytes was examined as the ratio of the number of positive cells to the total number of cells in a total of six locations, counting in two fields of observation per group.

### 2.3. Quantitative RT-qPCR Analysis of MMP-13 and Nrf2

The expression levels of *MMP-13*, which is associated with OA severity [19], and *Nrf2*, which is an inducer of antioxidant signaling, were evaluated [20,21]. Total RNA was isolated using an RNA extraction kit (Isogen; Nippon Gene, Tokyo, Japan) and subjected to DNase digestion. cDNA was synthesized using the High-Capacity RNA-to-cDNA Kit (Thermo Fisher Scientific) in a 20 μL mixture containing 1 μg total RNA. RT-qPCR analysis was performed using cDNA equivalent to 20 ng RNA in a 10 μL reaction volume on a QuantStudio 3 Real Time PCR System (Thermo Fisher Scientific). Gene analysis of human chondrocytes was performed using the TaqMan Fast Advanced Master Mix (Thermo Fisher Scientific) and one of the TaqMan gene expression assays (Thermo Fisher Scientific), namely *MMP-13* (Hs00233992_m1) or *Nrf2* (Hs00900735_m1), and normalized against the expression of *18S rRNA* gene (Hs03003631_g1). The threshold cycle (Ct) was determined after setting the threshold for the linear amplification step of PCR. The ΔCt for specific genes was defined as Ct (target gene) − Ct (18S). Data were analyzed using the relative quantification method (ΔΔCt) with the QuantStudio Design and Analysis software v1.5 (Thermo Fisher Scientific).

### 2.4. Determination of Viability of Cells

Cell viability was determined using the Cell Counting Kit-8 (CCK-8; Dojindo Laboratories, Kumamoto, Japan). Human chondrocytes were seeded in a 96-well plate at a density of 1.0 × 10^4^ cells/well and incubated for 24 h. Thereafter, 10 µL of CCK-8 solution was added to each well and the plate was incubated at 37 °C for 2 h. The absorbance was measured at 450 nm using an iMark microplate reader (Bio-Rad Laboratories, Inc., Hercules, CA, USA). The absorbance was also measured after 24 and 48 h of treatment.

### 2.5. Statistical Analysis

Quantitative data are expressed as a mean ± standard error of the mean (SE). Data from RT-qPCR and viable cells were analyzed using one-way ANOVA; *p*-values < 0.05 were considered statistically significant using the Dunnett’s test. IF data were analyzed by one-way ANOVA; *p*-values < 0.05 were considered statistically significant using the Bonferroni test. All quantitative data were analyzed using the EZR software version 1.68.

## 3. Results

### 3.1. Preparation and Conditioning of H_2_O_2_-Induced Chondrocyte Injury Model

**Figure 2:**
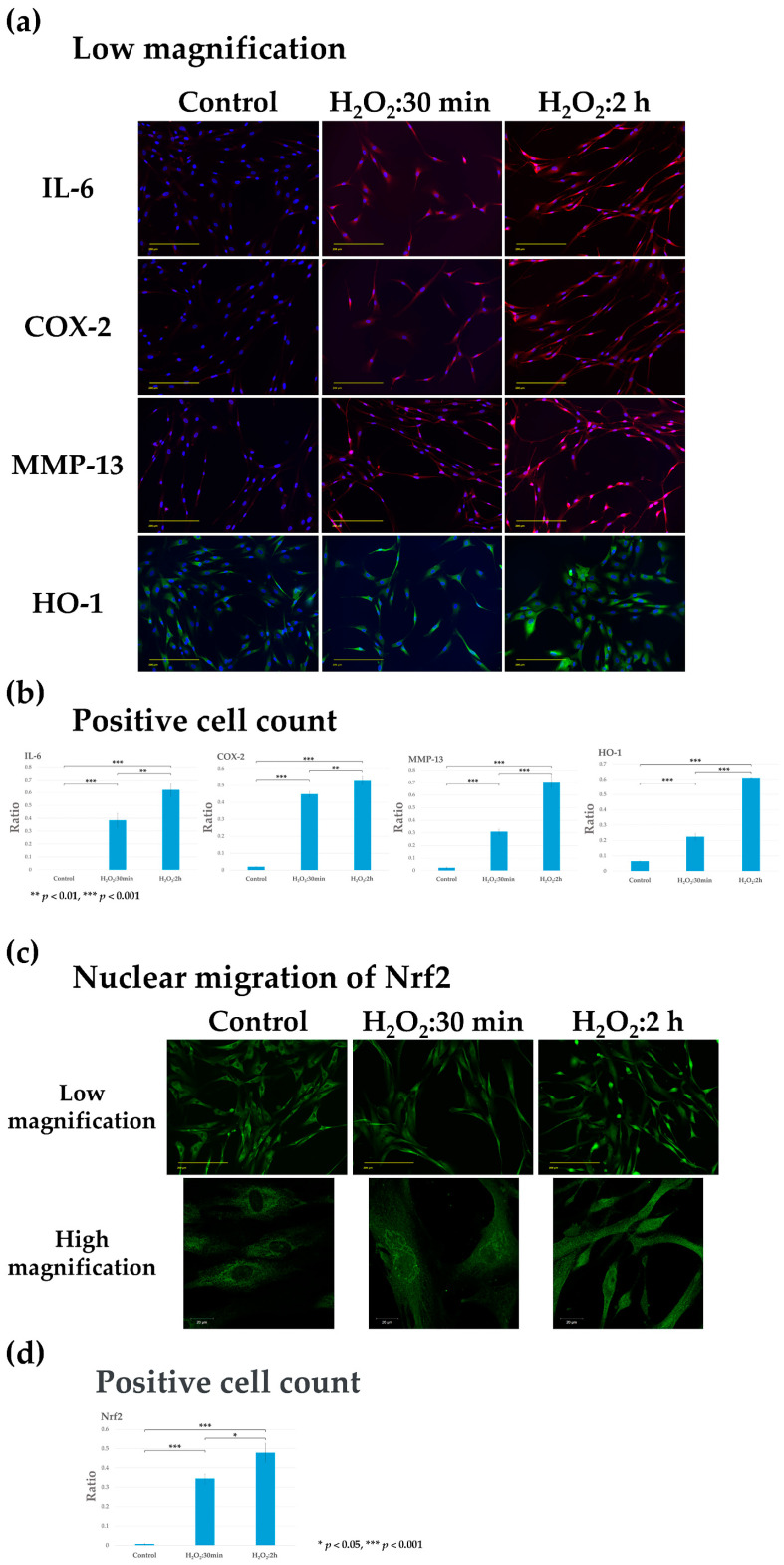
Fluorescence immunostaining of inflammatory (IL-6, COX-2, and MMP-13) and anti-inflammatory (Nrf2 and HO-1) factors in the human osteoarthritis-like chondrocyte model (human chondrocytes stimulated with H_2_O_2_ for 30 min or 2 h). Representative images showing IL-6, COX-2, and MMP-13 expression in red, Nrf2, and HO-1 expression in green, and DAPI-stained nuclei in blue. Scale bar, 200 μm. After stimulation of chondrocytes with H_2_O_2_ for 2 h, the expression of IL-6, Cox-2, and MMP-13 was increased; Nrf2 was expressed in the cytoplasm in controls and was translocated into the nucleus after 2-h H_2_O_2_ stimulation. HO-1 expression was decreased in the treated groups. (**a**) IL-6, Cox-2, MMP-13, HO-1; Low magnification. (**b**) Quantification of IL-6, COX-2, MMP-13, and HO-1 in chondrocytes was conducted by calculating the ratio of the number of cells positive for the protein expression to the total number of cells. Expression of IL-6, Cox-2, and MMP-13 were both significantly increased after 30 min and 2 h stimulation with H_2_O_2_ compared to control (*** *p* < 0.001). Compared with the 30 min stimulation with H_2_O_2_, there was a significant increase at 2 h (IL-6, COX-2; ** *p* < 0.01; MMP-13, HO-1; *** *p* < 0.001). (**c**) Nrf2; representative images in low magnification (Scale bar, 200 μm) and high magnification (Scale bar, 20 μm) are shown. (**d**) Quantification of the number of cells demonstrating nuclear migration of Nrf2. Nuclear translocation of Nrf2 was significantly increased at both 30 min and 2 h stimulated with H_2_O_2_ compared to control (*** *p* < 0.001). Comparing 30 min to 2 h showed a significant increase at 2 h (* *p* < 0.05).

Human chondrocytes were subjected to injury with 100 μM H_2_O_2_ for either 30 min or 2 h to create an in vitro OA chondrocyte model. The expression of inflammatory factors IL-6 and COX-2 were elevated in injured chondrocytes compared with that in the control. Moreover, the expression of MMP-13 and HO-1 was significantly increased in damaged chondrocytes at 2 h compared with that at 30 min (Figure 2a,b).

Nrf2 was localized in the cytoplasm of the control cells but migrated to the nucleus in chondrocytes exposed to H_2_O_2_ for either 30 min or 2 h. At 30 min, the expression of Nrf2 was observed both in the cytoplasm and nucleus; but at 2 h, the protein was predominantly localized in the nucleus (Figure 2c,d). The expression of HO-1 showed an increase concomitant with the nuclear migration of Nrf2.

Based on these results, it was concluded that 2 h exposure of chondrocytes to H_2_O_2_ was appropriate for evaluating the therapeutic effect of the COX-2 and IL-6 inhibitors on damaged cartilage using this model of human OA chondrocyte injury.

### 3.2. Pretreatment with COX-2- and IL-6 Inhibitors Suppresses Chondrocyte Injury

**Figure 3:**
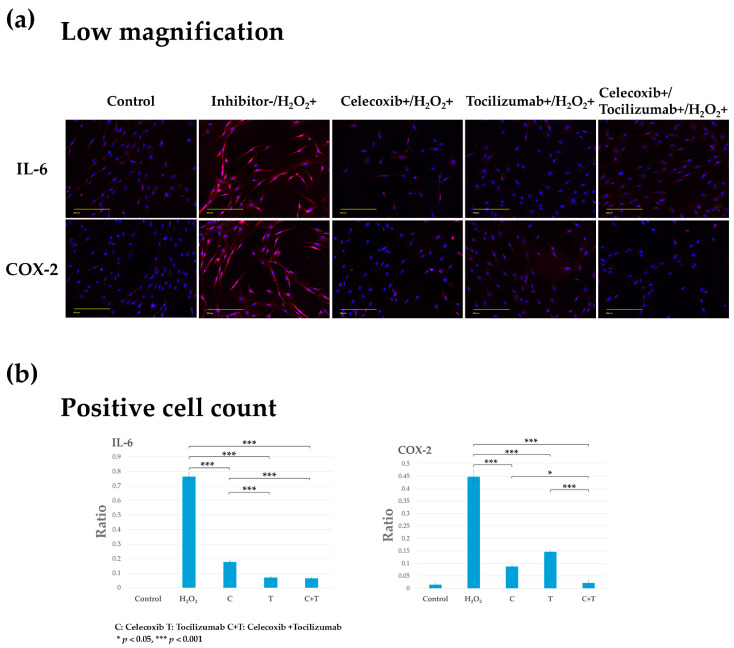
Fluorescence immunostaining of inflammatory factors (IL-6 and COX-2) in the human osteoarthritis chondrocyte injury model (human chondrocytes stimulated with H_2_O_2_ for 2 h) after pretreatment with celecoxib or tocilizumab alone or in combination for 24 h. Control: control group. Celecoxib+/H_2_O_2_-, Tocilizumab+/H_2_O_2_-, Celecoxib+/Tocilizumab+/H_2_O_2_-; Human chondrocytes pretreated with inhibitors alone or in combination for 24 h. Inhibitor-/H_2_O_2_+; Inflammation group, in which human chondrocytes were stimulated with H_2_O_2_ for 2 h. Celecoxib+/H_2_O_2_+, Tocilizumab+/H_2_O_2_+, Celecoxib+/Tocilizumab+/H_2_O_2_+; Prevention group, in which human chondrocytes were treated with inhibitors alone or in combination for 24 h, followed by stimulation with H_2_O_2_ for 2 h. (**a**) Representative images showing IL-6 and COX-2 expression in red and DAPI-stained nuclei in blue. Scale bar, 200 µm (Low magnification). (**b**) Quantification of IL-6 and COX-2 in chondrocytes was conducted by calculating the ratio of the number of cells positive for the protein expression to the total number of cells. The expression of IL-6 and COX-2 was upregulated and downregulated, respectively, in the inflammation and prevention groups. The expression of IL-6 and COX-2 was significantly diminished in all prevention groups in comparison to the inflammation group (*** *p* < 0.001). Comparing inhibitors alone to the combination showed a significant reduction in the combined (IL-6; *** *p* < 0.001 for C or T vs. C+T; COX-2; * *p* < 0.05 for C vs. C+T, *** *p* < 0.001 for T vs. C+T).

Chondrocytes were pretreated with celecoxib and tocilizumab, alone or in combination, for 24 h, followed by stimulation with H_2_O_2_ for 2 h. No COX-2 or IL-6 expression was observed after 24 h of treatment with these inhibitors. All treatment groups showed reduced IL-6 expression compared with that in the inflammation group, with a marked reduction in expression in the tocilizumab monotherapy and combination groups (Tocilizumab+/H_2_O_2_+, Celecoxib+/Tocilizumab+/H_2_O_2_+) compared with that in the H_2_O_2_ group (Inhibitor-/H_2_O_2_+) (Figure 3a). COX-2 was similarly downregulated in all treatment groups, with a marked reduction observed in the celecoxib monotherapy and combination groups (Celecoxib+/H_2_O_2_+, Celecoxib+/Tocilizumab+/H_2_O_2_+) compared with that in the inflammation group (Inhibitor-/H_2_O_2_+) (Figure 3b).

The significant suppression of COX-2 and IL-6 expression in the combination group confirmed that the combination of the two drugs did not inhibit their respective functions. Celecoxib exhibited inhibitory effects on both COX-2 and IL-6, whereas tocilizumab demonstrated similar inhibitory effects on both IL-6 and COX-2. This suggests that the suppression of either IL-6 or COX-2 has an inhibitory effect on the other.

### 3.3. Monotherapy and Combination Therapy with Celecoxib and Tocilizumab Are Effective in Treatment and Prevention of Progression in the Human Osteoarthritic Cartilage Injury Model

**Figure 4:**
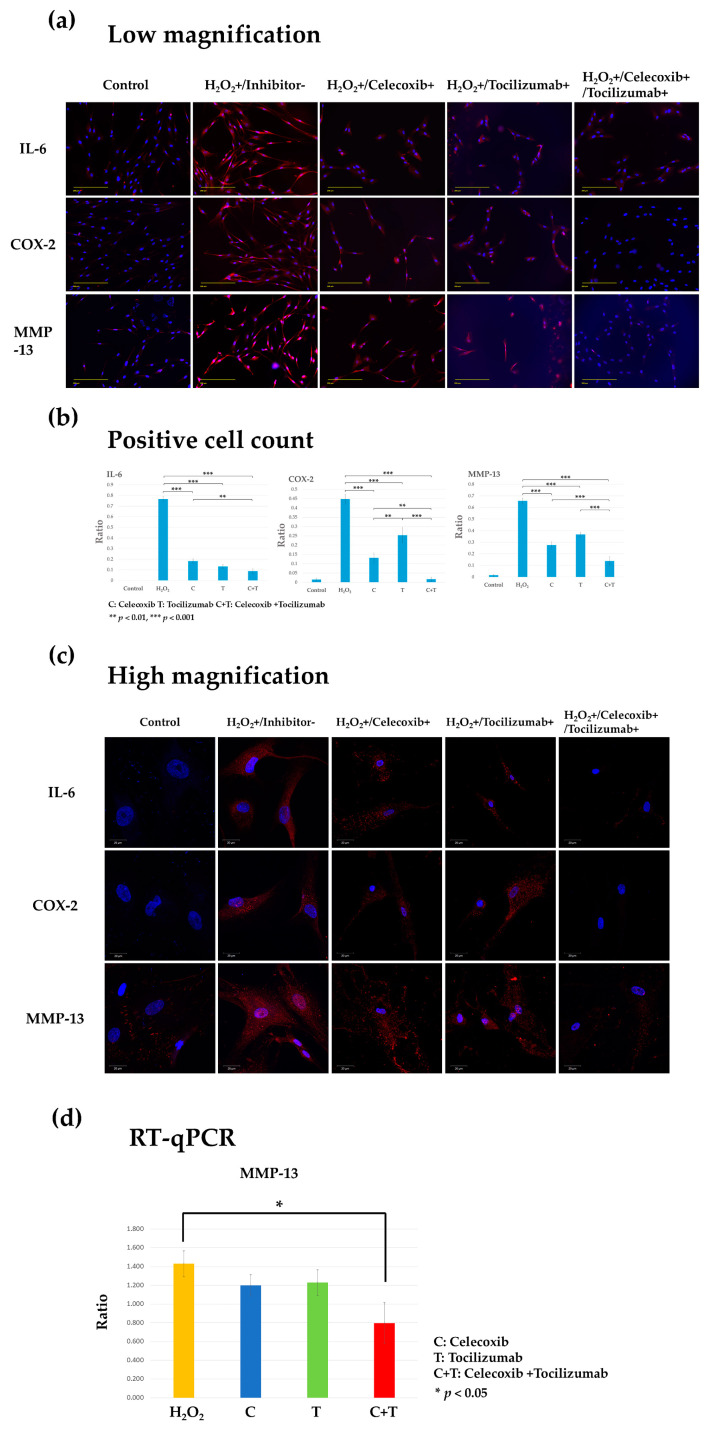
Fluorescence immunostaining and gene expression analysis of inflammatory factors (IL-6, COX-2, and MMP-13) in the human osteoarthritis chondrocyte injury model treated with celecoxib or tocilizumab alone or in combination. Control: control group. Inhibitor-/H_2_O_2_+: Inflammation group, in which human chondrocytes were stimulated with H_2_O_2_ for 2 h. H_2_O_2_+/Celecoxib+, H_2_O_2_+/Tocilizumab+, H_2_O_2_+/Celecoxib+/Tocilizumab+; Inhibitor-/H_2_O_2_+/Celecoxib+/Tocilizumab+; Inflammation group subjected to monotherapy or combination therapy with the inhibitors for 24 h. Representative images showing IL-6, COX-2, and MMP-13 expression in red and DAPI-stained nuclei in blue. (**a**) Low magnification (Scale bar, 200 µm). (**b**) Quantification of IL-6, COX-2, and MMP-13 in chondrocytes was conducted by calculating the ratio of the number of cells positive for the protein expression to the total number of cells. The expression of IL-6, COX-2, and MMP-13 was observed to be significantly decreased in all treatment groups that included inhibitors in comparison to the inflammation group that was stimulated with H_2_O_2_ (*** *p* < 0.001). A significant reduction was observed with the combination when compared to the inhibitors alone, with MMP-13 exhibiting the most notable decline (*** *p* < 0.001). (**c**) High magnification (Scale bar, 20 µm). (**d**) Gene expression analysis in inflammation and treatment groups. *MMP-13* expression was upregulated in the inflammation group and was significantly suppressed in the combination treatment with celecoxib (C) and tocilizumab (T): * *p* < 0.05 for H_2_O_2_ vs. C+T.

To determine the effects of both the drugs on chondrocyte damage, chondrocytes were stimulated with H_2_O_2_ for 2 h without drug pretreatment to induce cartilage damage. Subsequently, while the chondrocytes were stimulated with H_2_O_2_, celecoxib and tocilizumab were administered alone or in combination for 24 h—defined as the treatment period—to treat the damaged chondrocytes. Thus, the chondrocytes in the inhibitor-treated group were stimulated with H_2_O_2_ for 26 h. A significant reduction in IL-6, COX-2, and MMP-13 expression was observed in the combination group (H_2_O_2_+/Celecoxib+/Tocilizumab+) compared with that in the monotherapy groups (H_2_O_2_+/Celecoxib+ and H_2_O_2_+/Tocilizumab+) (Figure 4a,b). In the monotherapy group, nuclear shrinkage and loss of cell morphology were evident. By contrast, in the combination treatment group (H_2_O_2_+/Celecoxib+/Tocilizumab+), the nuclear and cell morphology were maintained (Figure 4c).

Gene expression analysis of *MMP-13* showed an increase in the H_2_O_2_ group (inhibitor-/H_2_O_2_+) and a decreasing trend in the treatment groups, with a significant decrease observed in the combination group (H_2_O_2_+/Celecoxib+/Tocilizumab+) (*p* < 0.05) (Figure 4d).

### 3.4. Therapeutic Effect of Celecoxib or Tocilizumab Is Mediated Through the Nrf2/HO-1 System

**Figure 5:**
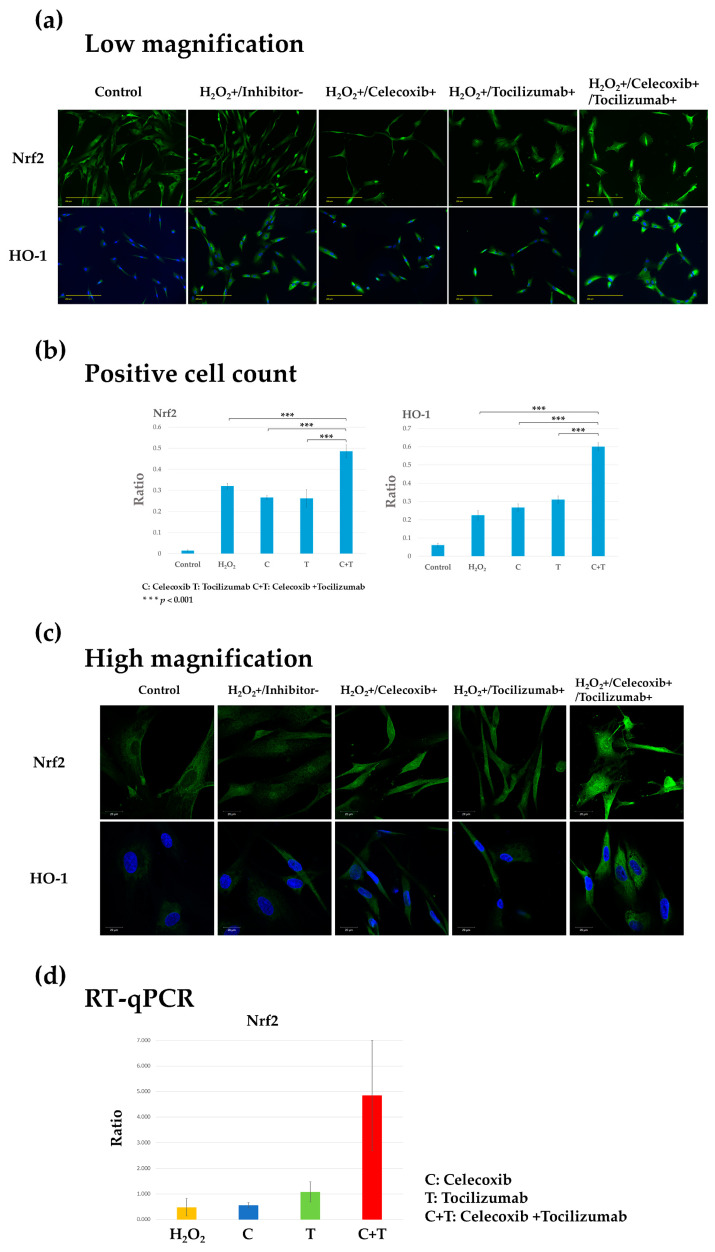
Fluorescence immunostaining and gene expression analysis of anti-inflammatory factors (Nrf2 and HO-1) in the human osteoarthritis chondrocyte injury model treated with celecoxib or tocilizumab alone or in combination. Control: control group. Inhibitor-/H_2_O_2_+: Inflammation group, in which human chondrocytes were stimulated with H_2_O_2_ for 2 h. H_2_O_2_+/Celecoxib+, H_2_O_2_+/Tocilizumab+, H_2_O_2_+/Celecoxib+/Tocilizumab+; Inhibitor-/H_2_O_2_+/Celecoxib+/Tocilizumab+; Inflammation group subjected to monotherapy or combination therapy with the inhibitors for 24 h. Representative fluorescence immunostaining images showing Nrf2 and HO-1 expression in green and DAPI-stained nuclei in blue. (**a**) Low magnification (Scale bar, 200 µm). (**b**) Quantification of HO-1 in chondrocytes was conducted by calculating the ratio of the number of cells positive for the protein to the total number of cells. Quantification of the number of cells demonstrating nuclear migration of Nrf2. The nuclear translocation of Nrf2 was significantly enhanced in the combined treatment group relative to the H_2_O_2_ stimulation group (*** *p* < 0.001). Nuclear translocation was also significantly greater with the combination than the inhibitors alone (*** *p* < 0.001). The expression of HO-1 was observed to be significantly increased in all treatment groups that included inhibitors in comparison to the inflammation group that was stimulated with H_2_O_2_ (*** *p* < 0.001). A significant increase was observed with the combination when compared to the inhibitors alone (*** *p* < 0.001). (**c**) High magnification (Scale bar, 20 µm). (**d**) Gene expression analysis in inflammation and treatment groups. *Nrf2* showed increased nuclear translocation in the treatment group compared with that in the inflammation group. In particular, an increasing trend was observed in treatment with a combination of celecoxib (C) and tocilizumab (T); *p* = 0.0623 for H_2_O_2_ vs. C+T.

As evident from the results described in previous sections, the combination of celecoxib and tocilizumab was more effective than either of the agents used alone. We further evaluated the efficacy of this combination. The expression of Nrf2 and HO-1 was markedly increased in the H_2_O_2_ group compared with that in the control group (Figure 5a,b). In the monotherapy group, although Nrf2 and HO-1 expression was observed, nuclear shrinkage and loss of cell morphology were evident. By contrast, in the combination treatment group (H_2_O_2_+/Celecoxib+/Tocilizumab+), the nuclei and cell morphology were maintained. Nuclear migration of Nrf2 was observed in all the treatment groups, with the combination group exhibiting the most notable results. Additionally, HO-1 levels induced by the nuclear migration of Nrf2 were significantly elevated in the combination group (Figure 5c). *Nrf2* gene expression showed an increasing trend in the combination group (H_2_O_2_+/Celecoxib+/Tocilizumab+) compared with that in the H_2_O_2_ group (H_2_O_2_+/inhibitor-) (*p* = 0.0623) (Figure 5d).

These results confirmed that the expression of proinflammatory factors (IL-6, COX-2, and MMP-13) was decreased, the expression of anti-inflammatory factors (Nrf2 and HO-1) was increased, and the cell morphology was preserved in the combination treatment group (H_2_O_2_+/Celecoxib+/Tocilizumab+), compared with that in the celecoxib and tocilizumab monotherapy groups (H_2_O_2_+/Celecoxib+ and H_2_O_2_+/Tocilizumab+).

### 3.5. Celecoxib and Tocilizumab Combination Restores Chondrocyte Viability

Cell viability of human chondrocytes stimulated with H_2_O_2_ for 2 h, followed by 24 h of treatment with celecoxib or tocilizumab monotherapy, or a combination of both, was assessed at 24 and 48 h after the treatment.

**Figure 6:**
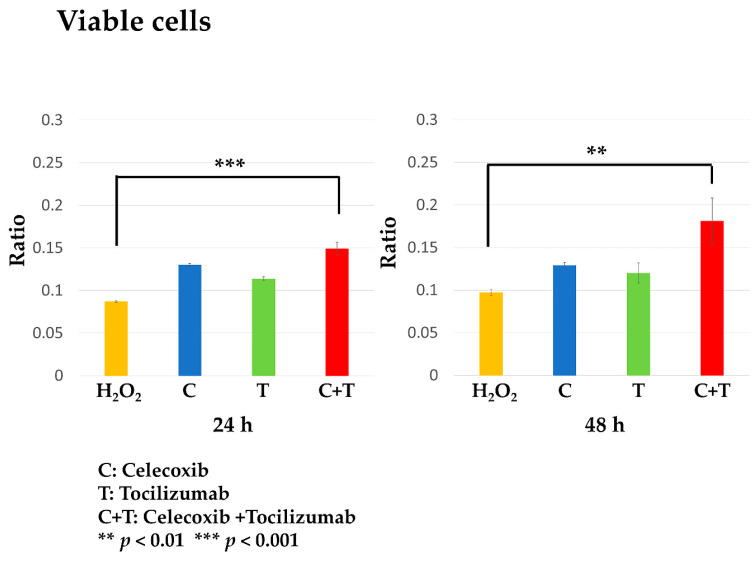
Analysis of cell viability in the human osteoarthritic cartilage injury model. Compared to the inflammation group, there was an increase in cell proliferation in the treatment group at 24 and 48 h after treatment, which was particularly significant in the combination group: H_2_O_2_ vs. C+T (24 h: ***, *p* < 0.001; 48 h: **, *p* < 0.01).

Compared to the H_2_O_2_ group (H_2_O_2_+/inhibitor-), all the treatment groups showed an increase in viable cell counts. In particular, in the combination groups (H_2_O_2_+/Celecoxib+ and H_2_O_2_+/Tocilizumab+), a significant increase in viable cell counts was observed after 24 and 48 h of treatment (24 h, *p* < 0.001; 48 h, *p* < 0.01) (Figure 6).

## 4. Discussion

Despite the long history of OA research, there is a dearth of effective therapeutic agents against this disease [22,23]. To investigate the efficacy of drugs already available clinically for the inhibition and treatment of cartilage damage, in the present study, we employed an OA-like chondrocyte injury model and two pharmacological agents—celecoxib, which is a COX-2 selective inhibitor, and tocilizumab, which is an anti-human IL-6 receptor monoclonal antibody that inhibits IL-6. We employed a multifactorial approach to evaluate the involvement of specific biomarkers in the pathogenesis of OA. These included IL-6, COX-2, and MMP-13, which are associated with inflammatory processes, and HO-1 and Nrf2, which are antioxidant factors. IL-6 is a target factor in OA cartilage, whose expression is downregulated by miR-653-5p, which inhibits chondrocyte senescence and alleviates cartilage degeneration via IL-6/JAK/STAT3 signaling [24]. COX-2 induces the synthesis of prostaglandin E2 (PGE2) using arachidonic acid as a substrate [25,26]; it is upregulated in inflamed joint tissue and is involved in increased production of PGE2 and other substances in OA joints [6]. COX-2 is also upregulated in chondrocytes and synoviocytes in OA [27]. It has also been directly implicated in abnormal subchondral bone remodeling, and cartilage and synovial inflammation via local regulatory mechanisms. In addition, MMPs play important roles in OA pathogenesis. Inflammatory responses and MMP activity influence the severity of OA by inducing cartilage degradation [19,28]. Furthermore, the siRNA-mediated knockdown of IL-6 in OA chondrocytes suppresses the expression and secretion of MMP-13; a direct link between IL-6 and MMP-13 expression has been reported [29]. Therefore, these factors were used as indicators of OA severity.

The activation of the Nrf2/HO-1 pathway has been reported to be effective in the treatment of OA, showing promise in suppressing NF-κB-mediated inflammatory effects [20,21]. Therefore, Nrf2 and HO-1 were used as indicators of factors that inhibit disability.

First, human chondrocytes were stimulated with 100 μM H_2_O_2_ for 30 min or 2 h [16] to establish the optimal conditions for the present study and were validated for the expression of inflammatory and anti-inflammatory factors. The levels of IL-6, COX-2, and MMP-13 increased when chondrocytes were stimulated with H_2_O_2_ for 30 min or 2 h. Stimulation of chondrocytes with H_2_O_2_ for 2 h significantly increased MMP-13 expression, which is associated with OA severity, and Nrf2 was activated in response to oxidative stress and showed nuclear translocation. In addition, the expression of HO-1, which is induced upon Nrf2 activation, was significantly increased. Therefore, in this study, human chondrocytes stimulated with H_2_O_2_ for 2 h were used as a model of OA-like cartilage damage to examine the preventive and therapeutic effects of celecoxib and tocilizumab, alone and in combination. The concentration of each drug was determined based on previous studies [17,18].

To investigate the effects of the drugs alone and in combination, we initially examined the pretreatment effects of celecoxib and tocilizumab alone and in combination on cartilage damage. IL-6 and COX-2, whose expression was confirmed by exposing chondrocytes to H_2_O_2_, were inhibited upon treatment with celecoxib and tocilizumab, respectively. This indicates the importance of inhibiting either IL-6 or COX-2 as a pretreatment. Therefore, it has been suggested that the use of either IL-6 or COX-2 inhibitors as a pretreatment in very early OA may reduce subsequent progression and inflammation. The inhibitory effect was maintained and enhanced in the combination group. These results indicated that the combination of the two drugs did not adversely impact the efficacy of the individual drugs.

In clinical practice, patients are typically observed after the onset of symptoms or after OA has progressed to a certain degree. Although the pretreatment effect is suitable for determining the efficacy of a drug, it is essential to consider its effect when evaluating the treatment as a whole. Consequently, for clinical prospects, it is essential to ascertain the impact of COX-2 and IL-6 inhibitors on the injured cartilage. Therefore, we conducted experiments to determine the effects of these drugs on chondrocytes after injury. H_2_O_2_ was retained throughout the treatment period to better represent the clinical characteristics of cartilage damage.

The expression of IL-6 and COX-2 was significantly decreased upon treatment with either drug. Therefore, both the drugs are expected to be slightly effective when used alone. This result was consistent with previous reports wherein inhibition of IL-6 by tocilizumab was shown to reduce the expression of COX-2 [30] and to attenuate the induction of STAT3-related genes, such as *COX-2* and *iNOS* [31]. In addition, increased expression of COX-2 has been shown to be associated with increased expression of inflammatory cytokines, and IL-6 expression has been reported to be reduced by COX-2 inhibitors [32] and COX-2 knockdown [33]. These reports suggest that each drug may have some anti-inflammatory effect, which is consistent with the results of the present study. The chondrocytes in the combination treatment group exhibited significantly reduced expression of COX-2 and IL-6. Similarly, a significant decrease in the expression of MMP-13, a factor related to OA severity, was observed in the combination group. Furthermore, a striking difference in cell morphology was observed between the monotherapy and combination therapy groups: the chondrocytes in the monotherapy groups showed loss of nuclear and cellular morphology, and nuclear shrinkage, whereas those in the combination therapy maintained the normal cell morphology and nuclear size. These results indicated that the combination of celecoxib and tocilizumab amplified the therapeutic effects of the individual drugs.

With regard to antioxidant factors, treatment with the celecoxib and tocilizumab combination resulted in pronounced enhancement of nuclear migration through the activation of Nrf2, accompanied by a simultaneous induction of HO-1, in comparison with that in the inflammatory group. In chondrocytes, Nrf2/HO-1 signaling inhibits NF-κB and markedly attenuates the inflammatory response and catabolism elicited by IL-1β and inflammatory cytokines, such as COX-2, PGE2, TNF-α, IL-6, and nitric oxide, by activating the Nrf2/HO-1 cascade [34].

Based on these reports, our findings suggest that celecoxib and IL-6 inhibitors may activate Nrf2 in injured chondrocytes, thereby promoting HO-1 induction and controlling inflammation. Furthermore, the number of viable cells, which was significantly decreased by H_2_O_2_ treatment, was significantly increased by the combination of celecoxib and tocilizumab compared with monotherapy. These findings indicate that the celecoxib and tocilizumab combination is an effective approach for the maintenance and recuperation of cartilage tissue in the post-treatment phase, consistent with prior investigations into the roles of inflammatory, anti-inflammatory, and antioxidative factors. As both pharmaceutical agents employed in this study have already been incorporated into clinical practice, their prospective clinical deployment may be relatively straightforward. Moreover, their combination may result in the maintenance and improvement of chondrocyte functionality after injury, without interfering with the effects of either drug, thereby potentially enhancing their applicability in the future management of OA.

This study had two major limitations. First, it is unclear whether the same results would be obtained in a cartilage damage model using other reported factors, given that this study used an osteoarthritis model generated via H_2_O_2_ exposure of chondrocytes. Second, considering this was an in vitro study, similar responses are not guaranteed in vivo. Thus, we believe that it is necessary to further investigate the use of previously reported factors that induce cartilage damage and to examine the results in animal models of OA.

## 5. Conclusions

This study shows that a combination of celecoxib and tocilizumab exerts sufficient anti-inflammatory effects against chondrocyte damage. Mechanistically, these inflammatory factors may be suppressed through the Nrf2-HO-1 pathway. We believe that the results of this study may improve therapeutic approaches against OA.

## Data Availability

The data sets used and/or analyzed during the current study are available from the corresponding author on reasonable request.

## References

[1] Deshpande B.R., Katz J.N., Solomon D.H., Yelin E.H., Hunter D.J., Messier S.P., Suter L.G., Losina E. (2016). Number of persons with symptomatic knee osteoarthritis in the US: Impact of race and ethnicity, age, sex, and obesity. Arthritis Care Res..

[2] Poulsen R.C., Jain L., Dalbeth N. (2023). Re-thinking osteoarthritis pathogenesis: What can we learn (and what do we need to unlearn) from mouse models about the mechanisms involved in disease development. Arthritis Res. Ther..

[3] Katz J.N., Arant K.R., Loeser R.F. (2021). Diagnosis and treatment of hip and knee osteoarthritis: A review. JAMA.

[4] Leistad L., Feuerherm A.J., Faxvaag A., Johansen B. (2011). Multiple phospholipase A2 enzymes participate in the inflammatory process in osteoarthritic cartilage. Scand. J. Rheumatol..

[5] Nah S.S., Choi I.Y., Lee C.K., Oh J.S., Kim Y.G., Moon H.B., Yoo B. (2008). Effects of advanced glycation end products on the expression of COX-2, PGE2 and NO in human osteoarthritic chondrocytes. Rheumatology.

[6] Sokolove J., Lepus C.M. (2013). Role of inflammation in the pathogenesis of osteoarthritis: Latest findings and interpretations. Ther. Adv. Musculoskelet. Dis..

[7] Lepetsos P., Papavassiliou A.G. (2016). ROS/oxidative stress signaling in osteoarthritis. Biochim. Biophys. Acta.

[8] Altindag O., Erel O., Aksoy N., Selek S., Celik H., Karaoglanoglu M. (2007). Increased oxidative stress and its relation with collagen metabolism in knee osteoarthritis. Rheumatol. Int..

[9] Wojdasiewicz P., Poniatowski Ł.A., Szukiewicz D. (2014). The role of inflammatory and anti-inflammatory cytokines in the pathogenesis of osteoarthritis. Mediat. Inflamm..

[10] Lepetsos P., Papavassiliou K.A., Papavassiliou A.G. (2019). Redox and NF-κB signaling in osteoarthritis. Free Radic. Biol. Med..

[11] He Y., Li Z., Alexander P.G., Ocasio-Nieves B.D., Yocum L., Lin H., Tuan R.S. (2020). Pathogenesis of osteoarthritis: Risk factors, regulatory pathways in chondrocytes, and experimental models. Biology.

[12] Liu Y., Zhang Z., Li T., Xu H., Zhang H. (2022). Senescence in osteoarthritis: From mechanism to potential treatment. Arthritis Res. Ther..

[13] Woolf C.J. (2011). Central sensitization: Implications for the diagnosis and treatment of pain. Pain.

[14] Yunus M.B. (2007). Fibromyalgia and overlapping disorders: The unifying concept of central sensitivity syndromes. Semin. Arthritis Rheum.

[15] Cheleschi S., Tenti S., Giannotti S., Veronese N., Reginster J.Y., Fioravanti A. (2021). A combination of celecoxib and glucosamine sulfate has anti-inflammatory and chondroprotective effects: Results from an in vitro study on human osteoarthritic chondrocytes. Int. J. Mol. Sci..

[16] Su C.Y., Luo Y., Fang C.H., Fang H.W. (2021). The effects of antioxidant supplements on the inflammatory gene expression of osteoarthritis-like chondrocytes. Appl. Sci..

[17] Cho H., Walker A., Williams J., Hasty K.A. (2015). Study of osteoarthritis treatment with anti-inflammatory drugs: Cyclooxygenase-2 inhibitor and steroids. BioMed Res. Int..

[18] Kuroyanagi G., Kamiya N., Yamaguchi R., Kim H.K.W. (2023). Interleukin-6 receptor blockade improves bone healing following ischemic osteonecrosis in adolescent mice. Osteoarthr. Cartil. Open.

[19] Bendele A.M., Neelagiri M., Neelagiri V., Sucholeiki I. (2021). Development of a selective matrix metalloproteinase 13 (MMP-13) inhibitor for the treatment of osteoarthritis. Eur. J. Med. Chem..

[20] Pan X., Chen T., Zhang Z., Chen X., Chen C., Chen L., Wang X., Ying X. (2019). Activation of Nrf2/HO-1 signal with myricetin for attenuating ECM degradation in human chondrocytes and ameliorating the murine osteoarthritis. Int. Immunopharmacol..

[21] Qu Y., Shen Y., Teng L., Huang Y., Yang Y., Jian X., Fan S., Wu P., Fu Q. (2022). Chicoric acid attenuates tumor necrosis factor-α-induced inflammation and apoptosis via the Nrf2/HO-1, PI3K/AKT and NF-κB signaling pathways in C28/I2 cells and ameliorates the progression of osteoarthritis in a rat model. Int. Immunopharmacol..

[22] Wieland H.A., Michaelis M., Kirschbaum B.J., Rudolphi K.A. (2005). Osteoarthritis—An untreatable disease?. Nat. Rev. Drug Discov..

[23] Bijlsma J.W.J., Berenbaum F., Lafeber F.P.J.G. (2011). Osteoarthritis: An update with relevance for clinical practice. Lancet.

[24] Lin Y., Zhang L., Ji M., Shen S., Chen Y., Wu S., Wu X., Liu N.Q., Lu J. (2024). MiR-653-5p drives osteoarthritis pathogenesis by modulating chondrocyte senescence. Arthritis Res. Ther..

[25] Chen C. (2010). COX-2′s new role in inflammation. Nat. Chem. Biol..

[26] Martel-Pelletier J., Pelletier J.P., Fahmi H. (2003). Cyclooxygenase-2 and prostaglandins in articular tissues. Semin. Arthritis Rheum..

[27] Mastbergen S.C., Lafeber F.P.J.G., Bijlsma J.W.J. (2002). Selective COX-2 inhibition prevents proinflammatory cytokine-induced cartilage damage. Rheumatology.

[28] Burrage P.S., Mix K.S., Brinckerhoff C.E. (2006). Matrix metalloproteinases: Role in arthritis. Front. Biosci..

[29] Haseeb A., Ansari M.Y., Haqqi T.M. (2017). Harpagoside suppresses IL-6 expression in primary human osteoarthritis chondrocytes. J. Orthop. Res..

[30] Chai E.Z., Siveen K.S., Shanmugam M.K., Arfuso F., Sethi G. (2015). Analysis of the intricate relationship between chronic inflammation and cancer. Biochem. J..

[31] Kinoshita H., Hirata Y., Nakagawa H., Sakamoto K., Hayakawa Y., Takahashi R., Nakata W., Sakitani K., Serizawa T., Hikiba Y. (2013). Interleukin-6 mediates epithelial-stromal interactions and promotes gastric tumorigenesis. PLoS ONE.

[32] Cheng Q., Li N., Chen M., Zheng J., Qian Z., Wang X., Huang C., Xu S., Shi G. (2013). Cyclooxygenase-2 promotes hepatocellular apoptosis by interacting with TNF-α and IL-6 in the pathogenesis of nonalcoholic steatohepatitis in rats. Dig. Dis. Sci..

[33] Zhu Y., Lu Y., Yuan L., Ling W., Jiang X., Chen S., Hu B. (2021). LincRNA-Cox2 regulates IL6/JAK3/STAT3 and NF-κB P65 pathway activation in *Listeria monocytogenes*-infected RAW264.7 cells. Int. J. Med. Microbiol..

[34] Jin J., Lv X., Wang B., Ren C., Jiang J., Chen H., Chen X., Gu M., Pan Z., Tian N. (2021). Limonin inhibits IL-1β-induced inflammation and catabolism in chondrocytes and ameliorates osteoarthritis by activating Nrf_2_. Oxid. Med. Cell Longev..

